# High-Speed Imaging of Amoeboid Movements Using Light-Sheet Microscopy

**DOI:** 10.1371/journal.pone.0050846

**Published:** 2012-12-05

**Authors:** Daisuke Takao, Atsushi Taniguchi, Takaaki Takeda, Seiji Sonobe, Shigenori Nonaka

**Affiliations:** 1 Laboratory for Spatiotemporal Regulations, National Institute for Basic Biology, Okazaki, Aichi, Japan; 2 Department of Life Science, Graduate School of Life Science, University of Hyogo, Kamigori-cho, Hyogo, Japan; 3 National Astronomical Observatory of Japan, Mitaka, Tokyo, Japan; University of Cambridge, United Kingdom

## Abstract

Light-sheet microscopy has been developed as a powerful tool for live imaging in biological studies. The efficient illumination of specimens using light-sheet microscopy makes it highly amenable to high-speed imaging. We therefore applied this technology to the observation of amoeboid movements, which are too rapid to capture with conventional microscopy. To simplify the setup of the optical system, we utilized the illumination optics from a conventional confocal laser scanning microscope. Using this set-up we achieved high-speed imaging of amoeboid movements. Three-dimensional images were captured at the recording rate of 40 frames/s and clearly outlined the fine structures of fluorescent-labeled amoeboid cellular membranes. The quality of images obtained by our system was sufficient for subsequent quantitative analysis for dynamics of amoeboid movements. This study demonstrates the application of light-sheet microscopy for high-speed imaging of biological specimens.

## Introduction

Selective plane illumination microscopy (SPIM) and its derivative, digital-scanned light-sheet microscopy (DSLM), were developed for fluorescence imaging of live biological specimens [1]–[6]. In SPIM, light-sheet illumination is achieved by selectively illuminate a single plane using a cylindrical lens [1]. The objective, which contains a CCD camera that acquires images, is placed with its optical axis perpendicular to path of illumination. The principle of DSLM is similar to that of SPIM except for the synchronization of the ‘apparent’ light-sheet by the scanning excitation beam and image acquisition [4]. The advantages of DSLM over SPIM include [4]: (1) a relatively constant intensity profile along the width of the ‘apparent’ light-sheet, which is important for quantitative imaging of large specimens; (2) a reduction in the optical aberrations; (3) an illumination efficiency of 95% for DSLM compared to approximately 3% for conventional SPIM. The major advantages of SPIM and DSLM are their efficiency of illumination, low phototoxicity, and high-speed image acquisition. In fact, the rate of image acquisition by SPIM is increased by 20 frames/s across a 360 × 360 pixel field of view [6].

Light-sheet microscopy is a very powerful tool for the biological studies. However, for many biologists who are not familiar with optics it is difficult to construct such an optical system on their own. In this regard, to simplify the system, we developed a DSLM that utilized the illumination optics from a conventional confocal laser-scanning microscope (CLSM). We referred to it as “ezDSLM” to reflect its ease of setup. The ezDSLM system makes light-sheet microscopy available to any laboratory because it is based on a conventional CLSM.

A shortcoming of conventional light-sheet microscopy is its limitations for high-speed imaging in some situations. For example, the specimen is typically embedded in agarose gel and moved through the light-sheet to obtain three-dimensional images. Since the position of light-sheet is fixed during imaging, the specimen must be moved using a sample holder. This can be problematic if the specimen needs to be observed without embedding in agarose gel, as is the case for the observation of the movements of amoeba on substrates or ciliary movements of cultured cells. Moreover, rapid movements or shaking of a specimen within medium may impact cell-substrate contacts or ciliary movements due to substrate vibrations or extracellular liquid flow. To overcome the limitations of standard light-sheet microscopy we modified the way that three-dimensional images are obtained by optically scanning the light-sheet instead of moving the specimen.

To demonstrate the ease of set-up for ezDSLM and its utility for biological imaging we observed the movements of *Amoeba proteus*. Although amoeboid movements can be observed by fluorescently labeling their plasmalemmas, live imaging has been limited to single optical planes. This was largely due to the fact that amoeba change their shapes too rapidly to obtain three-dimensional images by CLSM [7]. Despite being able to overcome this limitation with conventional DSLM, it would introduce the new problem of sample shaking as described above. Herein we describe the ezDSLM to capture clear images of rapidly moving amoeba with both high spatial and temporal resolutions that are sufficient for subsequent quantitative analyses.

## Results and Discussion

### Overview of the ezDSLM

**Figure 1:**
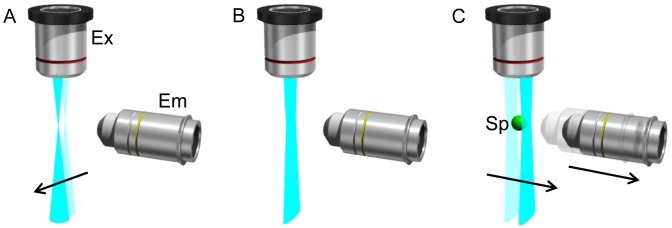
Schematic of ezDSLM (A, B). Line scan (A) to make an ‘apparent’ light-sheet (B). This step is similar to that in the conventional DSLM. (C) Scan of the light-sheet to obtain three-dimensional image of specimen. The objective for emission detection is synchronized with the movement of the light-sheet scanning. Ex, objective for excitation; Em, objective for emission detection; Sp, specimen.

We developed ezDSLM. Schematic drawings of image acquisition by ezDSLM are shown in Figure 1 and Movie S1. As in conventional DSLM [4], the line scan of a laser beam is used to generate a light-sheet that is synchronized with image acquisition by a CCD camera (Fig. 1A,B). In contrast to conventional DSLM, ezDSLM uses a two-dimensional scan of illumination to move the ‘apparent’ light-sheet along the axis of objective for emission detection. To keep the light-sheet on the focal plane of the objective for emission detection, the objective is mounted to the motor stage and synchronized to the ‘apparent’ light-sheet (Fig. 1C). Since it is unnecessary to move specimens during imaging, fast scans for three-dimensional image acquisition is achieved without shaking the specimens. Therefore, freely-moving specimens in medium can be observed without outside interference.

**Figure 2:**
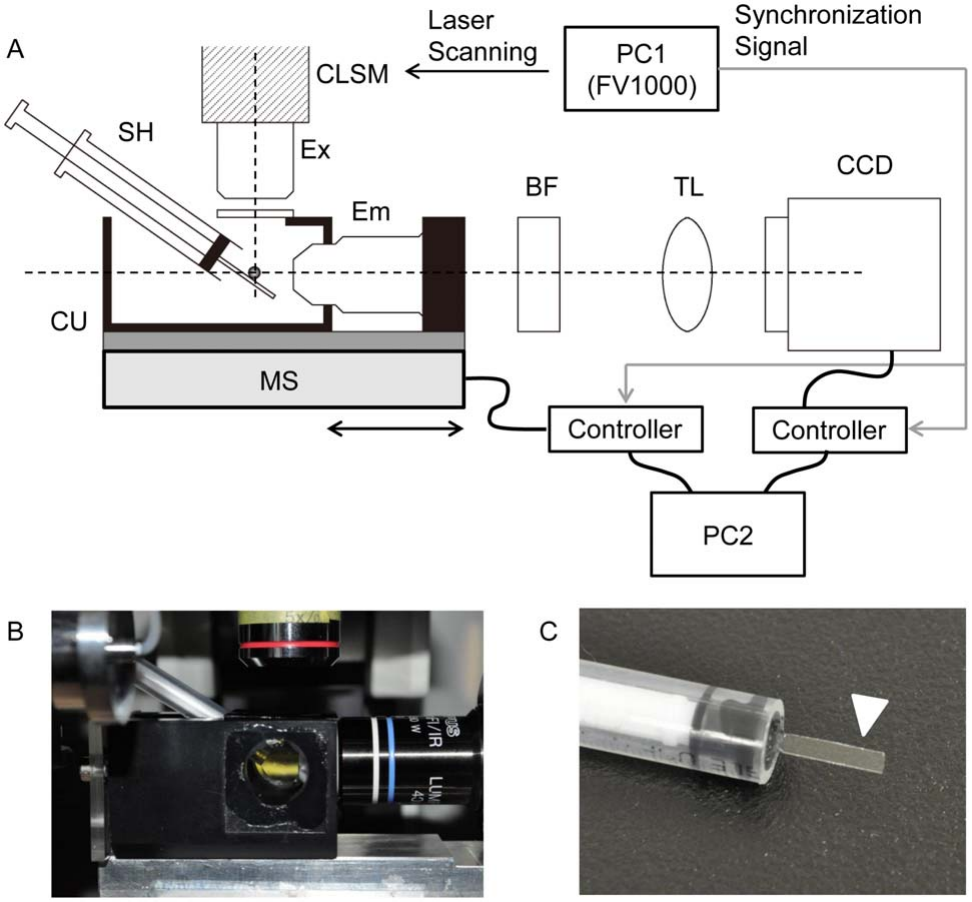
Setup of ezDSLM for observation of amoeboid movements. (A) Side view of the instrument (not to scale). Optical axes of objectives for excitation (Ex) and emission (Em) are shown in dashed lines. The illumination optics were derived from the confocal laser scanning microscope (CLSM). Specimens were placed on the sample holder (SH) in the chamber unit (CU) filled with medium. The CU containing the objective for emission detection is mounted on a movable motor stage. BF, barrier filter; TL, tube lens; CCD, CCD camera. (B) Photograph of the chamber unit. (C) Photograph of the sample holder. Amoebae were placed on the cover glass for imaging (arrow head).

To simplify the ezDSLM system we used the illumination optics from a CLSM system (Fig. 2A). The FV1000 system (Olympus) was used as a master of signaling and electric devices were fully synchronized (Fig. S1). Laser scanning for illumination is controlled by FV1000, which outputs electric signals for synchronization. The signals are then used to trigger movement of the objective mounted on the motor stage and image acquisition by the CCD camera. The ezDSLM system is very simple because it utilizes the illumination optics of the CLSM, which transforms it into a light-sheet microscope. It is also highly advantageous to biologists because it makes light-sheet microscopy available to people without special skills or expertise.

### Observation of Amoeboid Movements by ezDSLM

**Figure 3:**
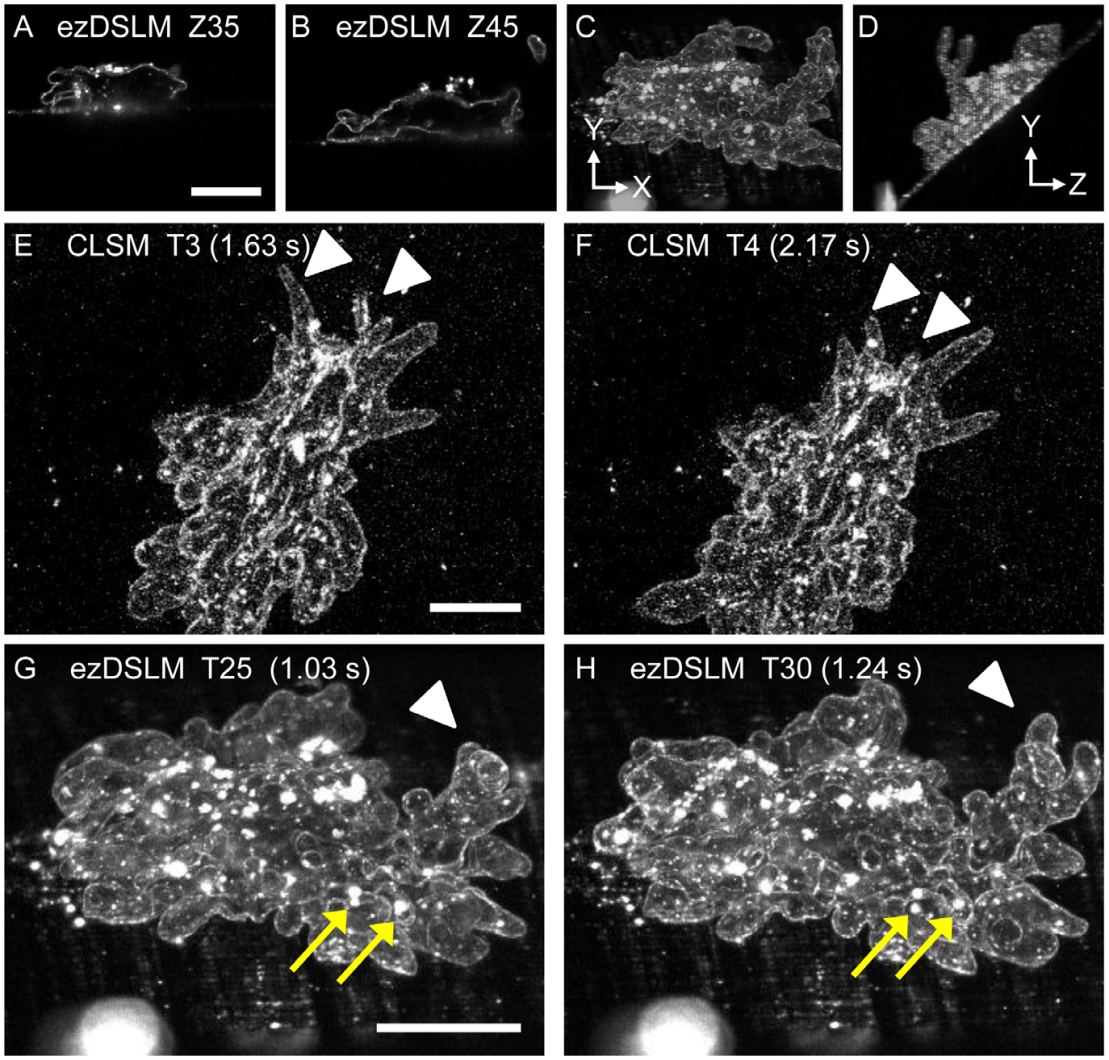
Images of freely moving DiI-labeled amoebae. Images were obtained using the ezDSLM (A–D, G, H) and CLSM (E, F). (A, B) X–Y optical sections from a Z-stack. Z35 and Z45 indicate slice numbers in Z-stacks with 2.48 µm intervals, i.e., actual distances are 86.8 and 111.6 µm, respectively. A complete series of Z-stack is shown in Fig. S2. (C, D) Front (C) and side (D) views of a reconstructed three-dimensional image. (E, F) Max projection images of Z-stacks at time-points T3 (E) and T4 (F) from time-lapse images obtained using CLSM. Intervals for each time-point were 0.54 s. It is difficult to follow the changes in shapes (arrowheads). (G, H) Max projection images from Z-stacks at time points T25 (G) and T30 (H) from time-lapse images obtained by ezDSLM. Intervals for each time-point were 0.04 s. Extensions of protrusions (arrowheads) and traceable clusters of dye (arrows) are depicted in detail. Note that the four more images (T26–T29) are present between these time points (not shown). Scale bars, 50 µm.

To evaluate the performance of ezDSLM, we applied it to high-speed imaging of amoeboid movements on substrates. A setup with standard conditions for the observation of the movements of *Amoeba proteus* [7] was constructed. Fluorescently labeled amoebae were placed on the glass surface of the sample holder (Fig. 2B,C). Amoebae have special membrane structures known as plasmalemma, which is composed of multiple layers including the plasma membrane, amorphous layer, and filamentous layer. The plasmalemma was labeled with lipophilic dye DiI. The chamber unit was filled with KCM medium, which is the standard medium for observation of amoebae. In conventional CLSM, it is difficult to observe the amoeboid movements with sufficient spatial and temporal resolutions for quantitative analyses. The images have low contrast and are blurred because amoebae change their shapes rapidly during the acquisition of images for a single XYZ stack. In ezDSLM, by contrast, we successfully obtained a time-series of XYZ-stacked images of whole amoebae (Fig. 3). The three-dimensional images clearly outlined the cell shapes of moving amoebae.

### Spatial and Temporal Resolutions Achieved by ezDSLM

The image quality, with regard to contrast or focusing, of ezDSLM was comparable to that in conventional DSLM, and thus ezDSLM worked well as a light-sheet microscope. The typical condition for image acquisition used here is as follows: Objectives for excitation, 5×, NA 0.10, air (Olympus), and for emission detection, 40×, NA 0.80, water dipping (Olympus); 336 × 256 pixels (corresponding to 197.0 × 228.2 µm) in XY resolutions; 2.48 µm intervals for each optical slice in Z stacks; frame rate, 40 frames/s. Under these conditions, it takes 2.5 s to acquire a Z stack of 100 X–Y slices (∼250 µm in Z dimension) that typically cover the whole body of a single amoeba (∼200 µm in Z dimension; Fig. 3D). Compared to the frame rate of the CLSM (2.39 frames/s with the same resolutions), ezDSLM achieved a 17-times higher rate. Although the frame rate of CLSM can be improved by using spinning disc or resonant scanner, light-sheet microscopy has advantages such as greater penetration depth of illumination and reduced photobleaching and phototoxicity, as described [1]–[6]. In addition, our observation of amoeba with the same preparation using a spinning disk microscope often produced false signal that was produced by out-of-focus-light passed neighboring pinholes (data not shown). The limiting step in our high-speed imaging system is the recording rate of the CCD camera. Therefore, the imaging rate of ezDSLM would be remarkably improved with a higher performance camera.

Using CLSM, the outlines of amoebae were frequently blurred since they moved rapidly during image acquisition. In addition, the displacement and changes in cell shapes of amoebae between each time frame were too large to follow and analyze (Fig. 3E,F, Movie S2). In contrast, images obtained with ezDSLM clearly outlined the cell shapes in -XYZ dimensions (Fig. 3A–D, Fig. S2, Movie S3), as well as in the time series of the Z-stack (Fig. 3G,H, Movie S4, Movie S5). Changes in cell shape, such as shrinkages and extensions of protrusions, were captured in detail with sufficient spatial and temporal resolutions. We also made time-lapse three-dimensional CG movies of membrane structures, using 4-dimensional digital universe (4D2U) system [8]. These movies depicted the motion of the membrane structures (Movie S6). Therefore, the image acquisition rate of ezDSLM was sufficiently high enough to follow three-dimensional shape changes of moving amoebae. Furthermore, clusters of accumulated dyes on the plasmalemma were traceable in the ezDSLM images (Fig. 3G,H). By using these clusters as landmarks, it is possible to analyze not only cell shapes but also membrane dynamics in detail.

### Quantitative Analysis

To show that ezDSLM is applicable for subsequent quantitative analyses, we measured dynamics of protrusions in amoeboid movements. Since protrusions extend three-dimensionally and changes in their shapes are rapid as described above, for precise analyses, three-dimensional images with both spatially and temporally sufficient resolutions are needed. In this regard, ezDSLM would be a suitable tool.

**Figure 4:**
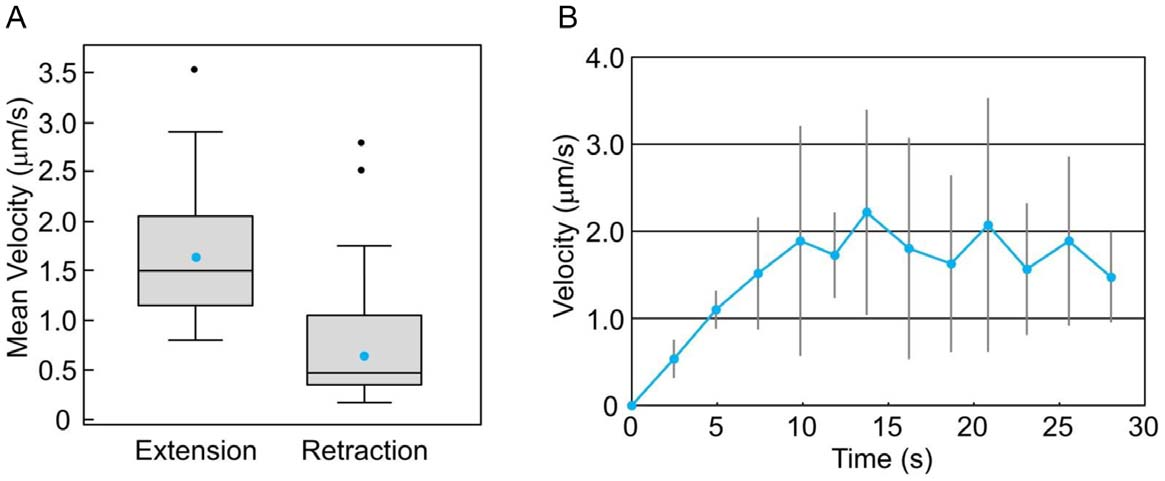
Dynamics of protrusions during amoeboid movements. By using the images obtained by ezDSLM, velocities of extending or retracting protrusions were measured. (A) Mean velocities of protrusions during extension or retraction are shown in box plots. Blue and black dots indicate mean values and outliers, respectively. The mean velocities of extension and retraction were significantly different by t-test (p<0.01). Data from 25 protrusions for each category are shown. (B) Time-evolved velocities of extending protrusions. Data from 5 protrusions are shown as mean±SD.

Using data obtained by ezDSLM, mean velocities of extension and retraction of protrusions were measured and compared (Fig. 4A). The mean velocity of extension was significantly higher than that of retraction, which coincides with the observations. We then measured time-evolved velocity of extension (Fig. 4B). Extensions of protrusions were accelerated for the early phase and they reached max velocities within ∼10 s. This may reflect the underlying mechanism in which actomyosin plays a role for extension of protrusion [9]–[12]. A small protrusion termed “bleb” is formed by contraction of the actomyosin cortex in the early phase, and this explosive force would accelerate the extension. Although further investigation is needed to understand biological significance, we showed that ezDSLM is applicable to such analyses.

### Biological Significance of Application of ezDSLM

In this study, we showed the application of ezDSLM for high-speed imaging and quantitative analysis of movements of *Amoeba proteus*. Amoeboid movements and membrane dynamics have been well characterized in dendritic cells [13] and leucocytes [14]. For *Amoeba proteus*, on the other hand, observation of cell movements has been difficult due to its high motility: ∼100 µm/min compared to <10 µm/min for fibroblasts. Therefore, imaging of motile *Amoeba proteus* has been limited to be two dimensions [7], [15]. Although some excellent studies of three-dimensional images of moving *Dictyostelium discoideum* were reconstructed from differential interference contrast (DIC) micrographs [16], [17], the contrast was relatively low compared to fluorescence images. In addition, traceable clusters of dyes were observable in cells stained with DiI (Fig 3G,H) making fluorescence imaging beneficial for the analyses of membrane dynamics. Collectively, high-speed imaging achieved by ezDSLM would serve as a powerful tool to investigate amoeboid movements as well as membrane dynamics. Further investigation using ezDSLM would provide a breakthrough in our understanding of the mechanisms of amoeboid movements. High-speed imaging with ezDSLM is also applicable to many other biological studies, such as the observation of ciliary movements. The most important features of ezDSLM include the observation of specimens without shaking and its ease of setup. These features of ezDSLM facilitate the application of light-sheet microscopy.

### Conclusions

We developed ezDSLM, which we used to capture remarkably clear images of moving amoebae. This process did not require the specimen to be shaken because the light-sheet is optically scanned to obtain three-dimensional images. We demonstrated the clarity and speed of ezDSLM to outline the movements of *Amoeba proteus*. The ezDSLM substantially extended the application of light-sheet-based microscopy in biological field. Finally, since our system uses a conventional CLSM system for illumination optics, setup is very simple and easily adaptable to other laboratories.

## Materials and Methods

### Cell Culture and Fluorescent Labeling of *Amoeba proteus*

*Amoeba proteus* was cultured in KCM culture medium (7 mg of KCl, 8 mg of CaCl_2_, 8 mg of MgSO_4_-7H_2_O per liter) at 25°C and fed on *Tetrahymena pyriformis* as previously described [18]. For live imaging of amoeboid movements the lyophilic dye DiI (Invitrogen) was used to label the plasmalemma. Amoebae were stained for 10 min in KCM medium containing 5.36 µM DiI (2.68 mM stock in DMSO) and washed three times with KCM medium. For imaging, the amoebae were placed on the sample holder (Fig. 2C) and rested for 15 min prior to imaging.

### Setup of ezDSLM

Setup of ezDSLM is schematically shown in Fig. 2, Fig. S1, and Movie S1. The Olympus FV1000 was used as the illumination optics as described above. The excitation wave length was 559 nm and the barrier filter BA570-625HQ (Olympus) was selected for detection of DiI. The motorized stage (M-111.1DG) and controller (C-862) for moving the objective were obtained from PI. The resolution of the stage is high (minimal incremental motion; 50 nm) and it has long travel range (15 mm). The movement of the stage was controlled by the original macros on the software, Mikromove (PI). The ORCA-R2 (Hamamatsu) was the CCD camera used for image acquisition. The camera was controlled by the HC Image software (Hamamatsu). The controllers were connected to a computer that is distinct from that in the FV1000 system. All electric devices were connected through their I/O interfaces and the FV1000 system was used as a master of synchronization signals. Parameters for laser scanning (laser power, scan rate, spatial interval, etc) were controlled by a computer in the FV1000 system. The parameters were adjusted for imaging conditions, e.g., the increments and range of movements for the light-sheet were typically 2.48 µm and 0.3 mm, respectively. When the FV1000 start scanning, it outputs synchronization signals that are active during each line scan (“line active” signal) or frame scan (“frame active” signal) as shown in Fig. S1. Exposures of CCD camera were synchronized to the line active signals so that each line scan makes apparent light-sheet. The motion of the motorized stage was triggered by the frame active signal and it moved at the constant rate that was synchronized to the movement of light-sheet. Trial-and-error processes may be required to adjust parameters at first, which can be achieved by imaging fluorescent microbeads or reflection of glass surface as in the case of conventional light-sheet microscopy. Images were processed using Image J, and in some cases, using the 4D2U system that is originally developed for astronomical studies [8].

### Measurements of Velocities of Protrusions

Coordinates (X, Y, Z) of extending or retracting edges of protrusions on the acquired images were manually tracked using ImageJ. By calculating displacements in each time interval, the velocities were obtained.

## Supporting Information

Figure S1 **Flow chart and waveforms of the synchronization signals.** The flow of the synchronization signals from the FV1000 is shown in upper panel. PC1 and PC2 are the computers that regulate laser scanning and other functions including image acquisition, respectively. PC1 is a master of the synchronization signal. CCD camera and motorized stage are regulated by PC2 through the controllers. Waveforms of the synchronization signals and responses of the devices are schematically shown in lower panel. “Line active” signal is active while the FV1000 scans lines to make the apparent light-sheet. “Frame active” signal is active during single movements of the apparent light-sheet.
(TIF)

Figure S2 **Complete series of Z-stack shown in** **Fig. 3A, B** **.** Slice numbers are labeled. Actual interval for each slice is 2.48 µm.
(TIF)

Movie S1 **Schematic animation of three-dimensional image acquisition by ezDSLM.** The objective for emission detection that is incorporated in the chamber unit is mounted on the motorized stage as shown in Fig. 2. The range of scanning is enlarged to clarify the detail of movement. Actual scanning range was typically small (∼0.3 mm) and the movement of the chamber unit did not affect amoeboid movements.
(MOV)

Movie S2 **Images of freely moving DiI-labeled amoebae, obtained using the CLSM.** Max projection images of Z-stacks were shown.
(MOV)

Movie S3 **Images of freely moving DiI-labeled amoebae, obtained using the ezDSLM.**
(MOV)

Movie S4 **Images of freely moving DiI-labeled amoebae, obtained using the ezDSLM.**
(MOV)

Movie S5 **Images of freely moving DiI-labeled amoebae, obtained using the ezDSLM.**
(MOV)

Movie S6 **Images of freely moving DiI-labeled amoebae, obtained using the ezDSLM.** The images were processed using the 4D2U system.
(MOV)
