# Supplementary figures and images for: High-Speed Imaging of Amoeboid Movements Using Light-Sheet Microscopy
Source: PLoS One. 2012 Dec 5;7(12):e50846. doi: 10.1371/journal.pone.0050846 (PMC3515486; doi:10.1371/journal.pone.0050846)

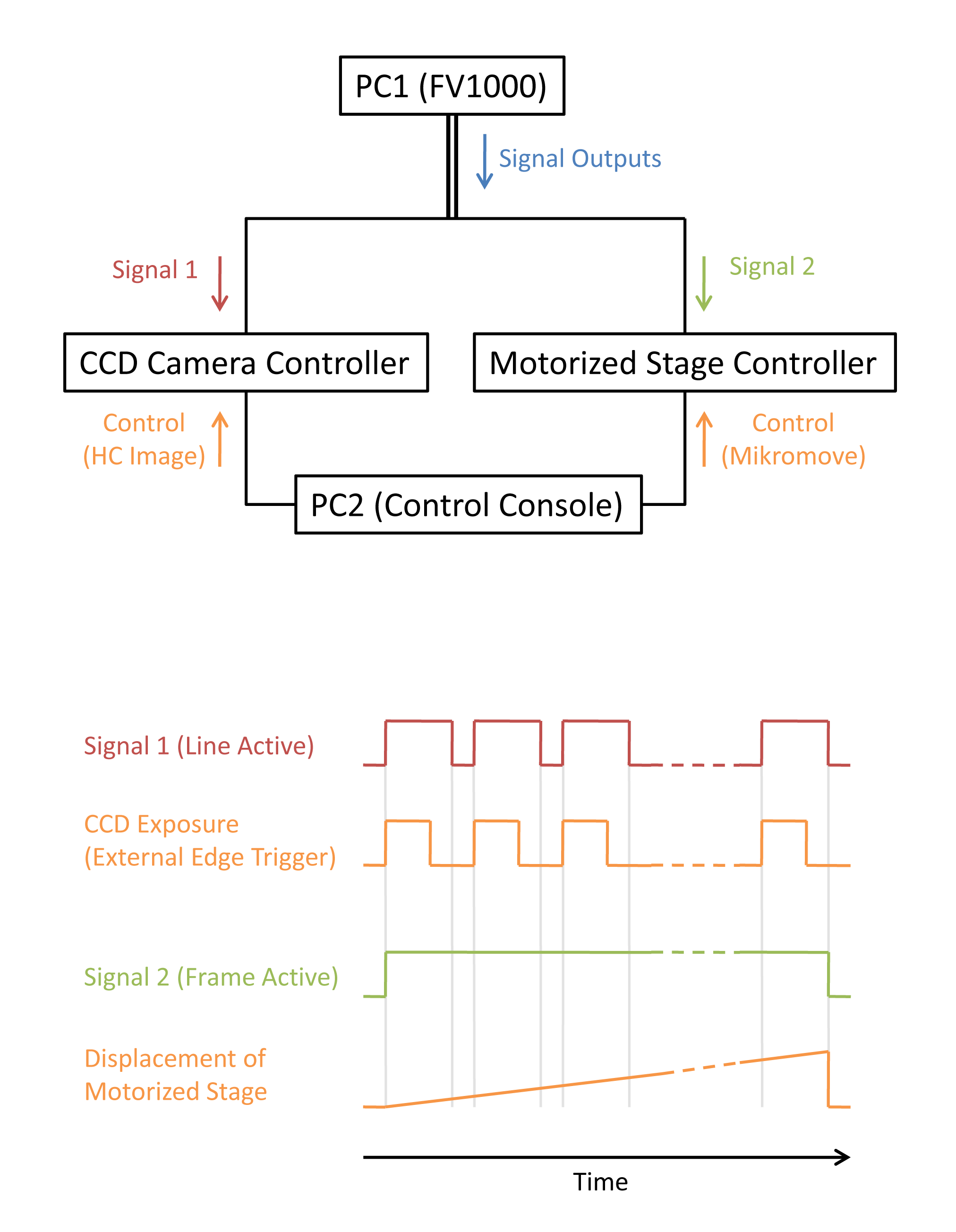

Supplement: Figure S1 — Flow chart and waveforms of the synchronization signals. The flow of the synchronization signals from the FV1000 is shown in upper panel. PC1 and PC2 are the computers that regulate laser scanning and other functions including image acquisition, respectively. PC1 is a master of the synchronization signal. CCD camera and motorized stage are regulated by PC2 through the controllers. Waveforms of the synchronization signals and responses of the devices are schematically shown in lower panel. “Line active” signal is active while the FV1000 scans lines to make the apparent light-sheet. “Frame active” signal is active during single movements of the apparent light-sheet. (TIF) [file pone.0050846.s001.tif]

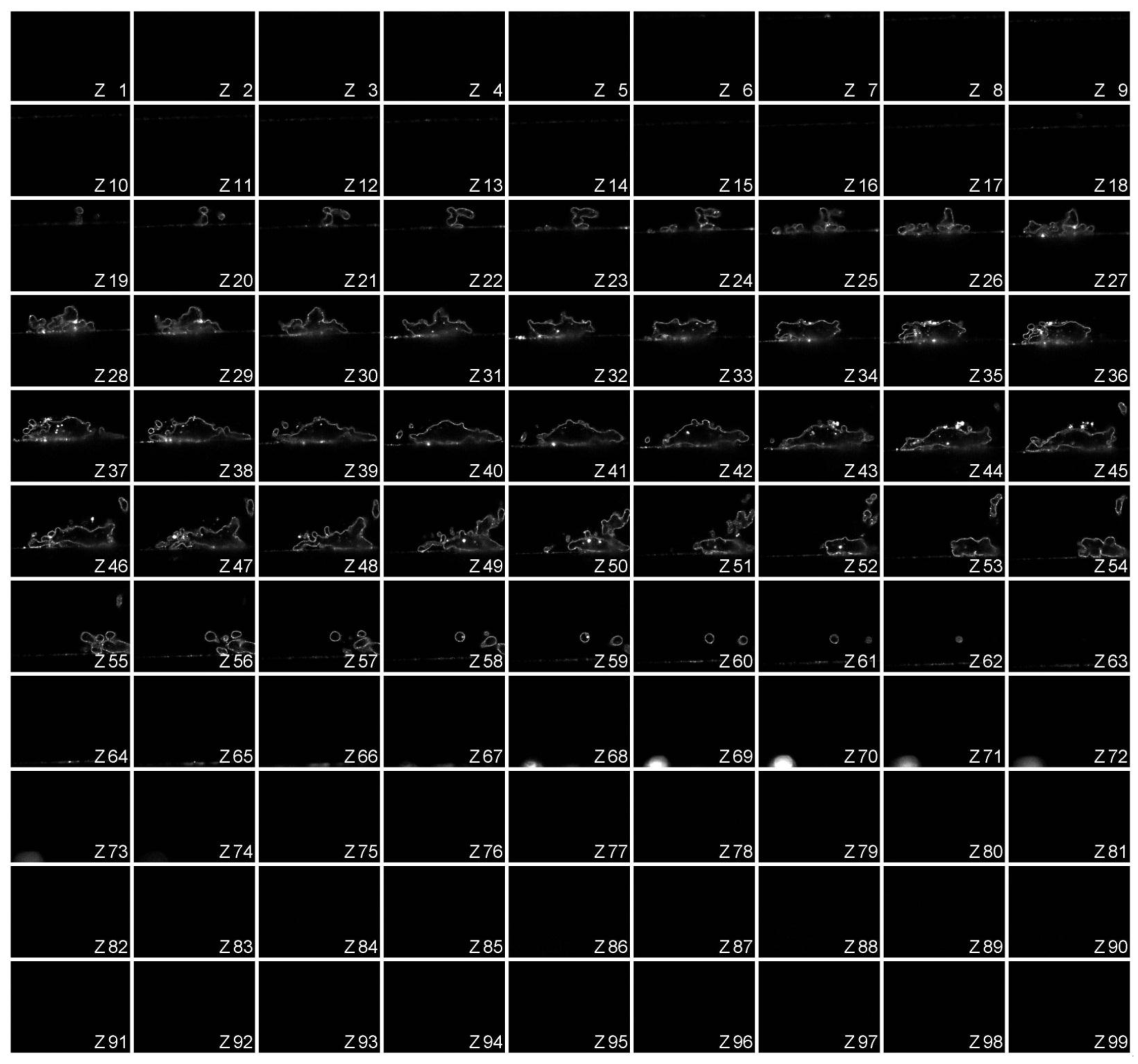

Supplement: Figure S2 — Complete series of Z-stack shown in Fig. 3A, B . Slice numbers are labeled. Actual interval for each slice is 2.48 µm. (TIF) [file pone.0050846.s002.tif]
